# A Novel Millet-Based Probiotic Fermented Food for the Developing World

**DOI:** 10.3390/nu9050529

**Published:** 2017-05-22

**Authors:** Elisa Di Stefano, Jessica White, Shannon Seney, Sharareh Hekmat, Tim McDowell, Mark Sumarah, Gregor Reid

**Affiliations:** 1 Food Microbiology, University of Wageningen, 6708 PB Wageningen, The Netherlands; elisa.distefano@wur.nl; 2 F3-106, Lawson Health Research Institute, 268 Grosvenor Street, London, ON N6A 4V2, Canada; jwhit44@uwo.ca (J.W.); shannon.seney@sjhc.london.on.ca (S.S.); 3 Food and Nutritional Sciences, Brescia College, London, ON N6G 1H2, Canada; hekmat@uwo.ca; 4 Agriculture and Agri-Food Canada, 1391 Sandford Street, London, ON N5V 4T3, Canada; Tim.McDowell@AGR.GC.CA (T.M.); mark.sumarah@agr.gc.ca (M.S.); 5 Departments of Microbiology & Immunology and Surgery, Western University, London, ON N6A 3K7, Canada

**Keywords:** probiotic, millet, yogurt, cereal, fermentation, sub-Saharan Africa

## Abstract

Probiotic yogurt, comprised of a Fiti sachet containing *Lactobacillus rhamnosus* GR-1 and *Streptococcus thermophilus* C106, has been used in the developing world, notably Africa, to alleviate malnutrition and disease. In sub-Saharan African countries, fermentation of cereals such as millet, is culturally significant. The aim of this study was to investigate the fermentation capability of millet when one gram of the Fiti sachet consortium was added. An increase of 1.8 and 1.4 log CFU/mL was observed for *S. thermophilus* C106 and *L. rhamnosus* GR-1 when grown in 8% millet in water. Single cultures of *L. rhamnosus* GR-1 showed the highest μ_max_ when grown in the presence of dextrose, galactose and fructose. Single cultures of *S. thermophilus* C106 showed the highest μ_max_ when grown in the presence of sucrose and lactose. All tested recipes reached viable counts of the probiotic bacteria, with counts greater than 10^6^ colony-forming units (CFU)/mL. Notably, a number of organic acids were quantified, in particular phytic acid, which was shown to decrease when fermentation time increased, thereby improving the bioavailability of specific micronutrients. Millet fermented in milk proved to be the most favorable, according to a sensory evaluation. In conclusion, this study has shown that sachets being provided to African communities to produce fermented milk, can also be used to produce fermented millet. This provides an option for when milk supplies are short, or if communities wish to utilize the nutrient-rich qualities of locally-grown millet.

## 1. Introduction

Malnutrition remains a major problem in many developing countries, especially in rural areas of sub-Saharan Africa [1,2]. An average of 98 deaths per 1000 live births occur in children under the age of five, which is 15 times more than in developed countries [3]. Underweight children are more susceptible to the “malnutrition-infection cycle“, as a result of a compromised immune system, predisposing them to infectious diseases, and increasing their risk of mortality [4,5,6]. The United Nations Standing Committee of Nutrition revealed that an essential factor in the nutritional status of young children is the quality of complementary foods [2,7].

Fermentation, such as of cereals, is an ancient and inexpensive food preservation method and a cultural and traditional practice within indigenous communities in Africa and in most developing countries [8]. It improves the nutritional value and digestibility of raw products (cereals, roots), enhances sensory characteristics, and improves the functional qualities available to local communities [8,9]. Traditionally, fermented foods and beverages (sour porridges, beverages, fermented vegetables, fruits, milk, meat, alcoholic and non-alcoholic beverages) represent a major dietary component in these countries [10,11,12]. The microorganisms may be indigenous to the food, or may be added as a starter culture after pre-treating or cooking the product [13]. The use of lactic acid bacteria (LAB) increases the acidity and decreases the pH of the substrate, thereby inhibiting many pathogens [14,15,16]. A number of LAB are used as probiotics, defined as “live microorganisms which, when administered in adequate amounts, confer a health benefit on the host” [17].

Cereal based foods contribute over half of the global food produced, and they are grown in over 73% of the world [14]. They are comprised of carbohydrates (60–70%), proteins (7–11%), fat (1.5–5%), crude fiber (2–7%), minerals and vitamins [18]. Proteins found in cereal are generally low quality compared to animal based proteins, as a result of a lower amount of some essential amino acids, such as threonine, lysine, and tryptophan [19]. The presence of anti-nutrients such as phytic acid, tannins, and polyphenols, can also bind to proteins, leading to a reduction in digestibility [11]. Fermentation by LAB has been shown to reduce phytic acids and tannins, therefore enhancing protein availability and digestion in various cereals such as maize, sorghum and finger millet [20,21]. Moreover, such fermented cereals have a higher composition of riboflavin, thiamine, niacin, and lysine [22,23].

Millet, a small seeded grain in the family Poaceae, is globally recognized as the sixth most important grain. It can survive desert-like conditions in tropical regions of Asia and Africa, is resistant to disease and pests, has a relatively short growing season, and is able to grow in less fertile soil conditions [18]. This grain is an example of a staple food for people of lower socioeconomic status. Nutritionally, millet is equivalent to other cereal grains, and has health promoting effects [18,24]. Pearl Millet (*Pennisetum glaucum*) is the major type of millet harvested in the world, accounting for 46%, followed by foxtail, proso and finger millet [18]. However, some communities have discontinued the fermentation of miller in favor of the cheaper and less labor-intensive maize [18].

Probiotics are essentially non-existent within poorer communities of Africa, but an initiative to introduce probiotic yogurt to Uganda, Tanzania and Kenya has led to the creation of one gram sachets [25]. These sachets are sourced from the Yoba for Life Foundation and contain one gram of dried starter culture of *Streptococcus thermophilus* and probiotic strains *Lactobacillus rhamnosus* GR-1 (Fiti), or a generic version of *L. rhamnosus* GG (Yoba) [26]. Each sachet can produce 100 L of probiotic yogurt, with a colony-forming unit (CFU)/mL of at least 1 billion, and is currently consumed by over 100,000 people per day in these countries. As milk is not always readily available, or is expensive in certain communities, we decided to test whether the sachet organisms could ferment millet with and without the addition of milk. Sensory characteristics and other properties that might make this a potential addition to health-promoting foods for the region were assessed and analyzed.

## 2. Materials and Methods

### 2.1. Cereal Matrix Preparation

Hulled pearl millet (*Pennisetum glaucum*) harvested from western USA was obtained from the Arva Flour Mill in Arva, Ontario, and whole grain pearl millet was obtained from the Agriculture Environmental Renewal Canada (AERC) Inc. in Ottawa, ON, Canada. Four main formulations were optimized: (i) water-based; (ii) milk-based; (iii) dried millet; and (iv) flour-based millet.

#### 2.1.1. Water-Based Millet

**Figure 1:**
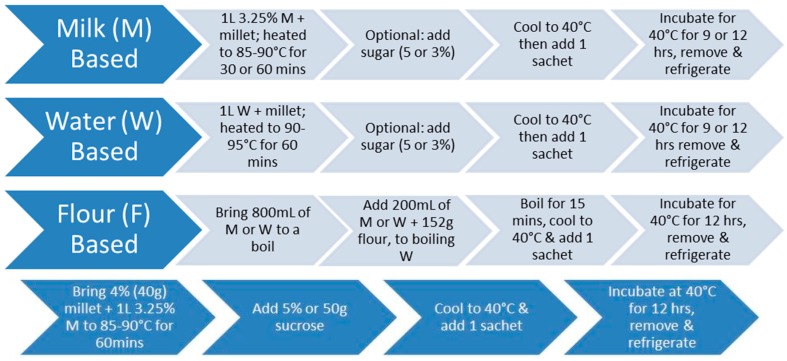
(**top**): Optimized preparation method for flour/milk/water based fermented millet products; (**bottom**): optimized recipe for 4% millet in milk, 60-min pre-treatment, 12 h of fermentation.

To prepare water-based fermented millet (Figure 1), one liter of water was added to the experimental concentrations of 4%, 6%, 7%, 8%, and 10% hulled millet. To degrade the starch in the millet, the mixture was brought to 90–95 °C for 60 min. Optionally, 5% sugar (sucrose or honey) was added in the last five minutes of the pre-treatment. After the pre-treatment, the mixture was cooled to 40 °C before adding the bacterial cultures.

#### 2.1.2. Milk-Based Millet

To prepare milk-based fermented millet (Figure 1), the same procedure was followed except that one liter of 3.25% homogenized milk, or 50% water and 50% milk, was added to the experimental concentrations of 3%, 4%, 6%, 7%, 9%, and 10% hulled millet. The mixture was brought to 85–90 °C and maintained for 30 or 60 min. After the pre-treatment, the mixture was cooled to 40 °C, before adding the bacterial cultures.

#### 2.1.3. Dried Millet

To prepare dried millet (Figure 1), one liter of water was added to 400 g of hulled pearl millet in a 2:1 ratio of water: millet. The mixture was then brought to a boil and reduced to low heat, covered and left to simmer until the water was absorbed. After the pre-treatment, the mixture was cooled to 40 °C, before adding the bacterial cultures.

#### 2.1.4. Flour-Based Millet

**Figure 2:**
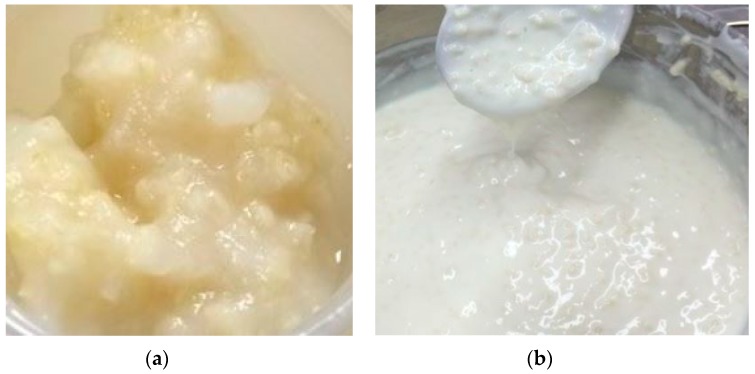
(**a**) 8% millet in water, 5% sucrose, 60-min pre-treatment, 12 h fermentation (40 °C); (**b**) 4% millet in milk, 60-min pre-treatment, 12 h fermentation (40 °C).

To prepare flour based millet (Figure 1), one liter of either milk, water, or 50% water and 50% milk was used. In a bowl, 200 mL of 3.25% homogenized milk was added to 152 g (one cup) of millet flour—obtained by using a blender to grind the whole grain into a flour—and then mixed. Subsequently, 800 mL of milk was added and brought to a boil. Once boiling point was reached, the wet flour mixture was added and stirred constantly at a boil for 15 min. After the pre-treatment, the mixture was cooled to 40 °C, before adding the bacterial cultures. Likewise, the same procedure was applied for the water-based mixture, and 50% water to 50% milk. Figure 2 illustrates the resultant fermented millet with milk.

### 2.2. Probiotic Strains and Preparation of Inocula

This was done to examine the growth properties of the strains, as the millet preparation was achieved by directly using the bacterial contents of the sachet.

### 2.3. Production of Probiotic Fermented Products

After cooling the cereal preparations to 40 °C, the Fiti sachet containing one gram of *L. rhamnosus* GR-1 and *S. thermophilus* was added to one liter of each, transferred to pre-sterilized jars, and incubated for 24 h at 40 °C, with sampling at intermediate times.

### 2.4. Determination of Acidity

A 25 g sample was titrated with 0.1 N NaOH according to the method described by Settachaimongkan et al. [27].

### 2.5. Bacterial Growth

For each food sample, 0.1 g was weighed and phosphate buffered saline (PBS) dilutions were then plated in triplicate on selective plates; LM17 was used to determine *S. thermophilus*, and MRS for *L. rhamnosus* GR-1. The plates were incubated anaerobically at 37 °C for 24 to 48 h. Viable counts were determined as CFU per mL, and an average count was recorded. Cell density of single cultures was assessed by the optical density measurement using a Multiskan Ascent plate reader (Thermo Fisher Scientific Inc., Waltham, MA, USA). Then, 200 μL of appropriately prepared sample was aliquoted into a 96 well-plate and the cell density was read at OD_600_. The same procedure was used to determine the utilization capacity for the strains with dextrose, fructose, galactose, lactose, maltose, melibiose, raffinose, and sucrose in M17 or modified MRS broth, at 37 °C for 24 h.

### 2.6. Sensory Evaluation

Two sensory analyses were conducted. Panel Test (A) consisted of 19 untrained participants, mainly Canadians, from Lawson Health Research Institute in London Ontario. Panel Test (B) consisted of three untrained participants from sub-Saharan African countries, studying at Western University in London Ontario. The purpose was to determine the ideal formulation or recipe that would be accepted by consumers. The test included varying products based on the cereal pre-treatment, fermentation (9 h vs. 12 h), amount of added sucrose (none vs. 3% or 5%), and the overall acceptance of milk or water based cereal. The first part of the evaluation involved a nine-point hedonic scale which allowed participants to rank samples from one (disliked extremely) to nine (liked extremely). In the second part, two of the same products but with differing pre-treatment and fermentation times were compared for texture, sweetness, acidity, appearance and overall acceptability. The samples used in the sensory analysis consisted of the 4% hulled pearl millet and milk, along with 8% and 10% hulled pearl millet and water. These concentrations were selected as a result of an in-house sensory analysis performed while testing various formulations. The yogurt to be sampled was made prior to the analyses and was stored in a refrigerator in a labeled jar according to its respective three-digit code. A tray was divided into two parts; cups were filled and labeled with their respective three-digit code. A water cup was placed in the middle of the tray to cleanse the palate between samples. A questionnaire was also provided to complete during the sensory testing. At Lawson, the analysis was held in an air conditioned, well-lit room. Participants were invited to complete the analysis during their lunch break in which they sat at an open table. They were asked to shield their work and to remain silent to avoid influencing other participants. With the African participants, the same protocol applied, however the environment differed as we conducted the analysis at various locations on Western University’s campus, and therefore we had to transport the trays and samples to where the participants were located (they did not all take part at the same time). Additionally, an in-person interview was conducted afterwards with the African participants, to assess the potential acceptance of the product in sub-Saharan countries (where the Fiti-Yoba kitchens are located), along with potential technological problems with respect to production, due to limitations in storage capacity and the availability of ingredients.

### 2.7. Metabolite Analysis

To extract metabolites such as organic acids and amino acids, a modified method from Urbaniak C et al. was used [28]. Firstly, 0.6 g of sample (milk, millet fermented in milk, millet fermented in water) were mixed with 800 µL of methanol and 200 µL of water (milk and millet fermented in milk samples), or 700 µL methanol (millet fermented in water samples). For each tested condition, two biological replicates were extracted and run three times (technical replicates). Samples were vortexed for 15 s and sonicated for 15 min at 35 °C in a Cole Parmer 8891 ultrasonic cleaner. Samples were centrifuged for 10 min at 9000× *g*. Supernatants (200 µL) were transferred to gas chromatography-mass spectrometry (GC-MS) vials and 2.5 µL of ribitol solution (2 mg/mL) was added to each vial as an internal standard. Samples were entirely dried using a SpeedVac (Labconco, Kansas City, MO, USA). For derivatization after drying, 100 µL of 2% methoxyamine hydrochloride in pyridine (MOX) was added to each sample and incubated at 50 °C for 90 min. *N*-methyl-*N*-(trimethylsilyl) trifluoroacetamide (100 μL) was then added to each vial and incubated at 50 °C for 30 min. Samples were analyzed on GC-MS (Agilent 7890A GC, 5975 inert MSD with triple axis detector (Agilent Technologies Inc., Wilmington, DE, USA), 30 m DB5-MS column with 10 m duraguard column) (30 m × 0.25 mm × 0.25 µm, Agilent, Folsom, CA, USA) using 2 µL injections on scan mode, and a solvent delay of 8.25 min. Run time was 61 min per sample.

Standard solutions were prepared for amino acids, acetate, 1,2-butandiol, citric acid, formate, fructose, galactose, glycerol, glucose, lactate, maltose, palmitic acid, stearic acid, succinic acid, and sucrose.

GC-MS chromatogram files were converted to ELU format using the AMDIS Mass Spectrometry software [29]. All chromatograms were aligned to each other, and the abundance of metabolites calculated using the Spectconnect software with the following settings: elution threshold: med (1 min), support threshold: med (≥75% of smps), similarity threshold: med (80% match), library similarity: med (80% match) [30], in order to determine whether differences between unfermented and fermented millet in water dispersion existed (0, 6, 12 h). Values in the relative abundance matrix that were equal to zero were replaced by two-thirds of the lowest non-zero value recorded for each sample. Following this, metabolite data were normalized to the internal standard for each sample and log(x + 1) transformed for principle component analysis (PCA) in MINITAB statistical software (State College, PA, USA).

Phytic acid concentration was determined by a modified Haug and Lantzsch method as described in Dietterich et al. [31,32]. Samples were extracted as per Wu et al. [33]. Briefly, approximately 1 mL of sample was mixed with 10 mL of 0.2 M HCl and incubated with rocking for 90 min at room temperature. The suspension was spun for 10 min at 2800 g and the supernatant was collected. The pellet was washed twice with 5 mL 0.2 M HCl, and the resulting supernatants were combined and made up to 25 mL with 0.2 M HCl. The assay was prepared by taking 25 μL of the sample extract or standard, adding 225 μL of 0.2 M HCl and 500 μL of iron solution, and boiling the sample for 30 min. Tubes were cooled to room temperature and centrifuged for 4500× *g* for 20 min. A 100 μL aliquot of this precipitation supernatant was pipetted into a 96 well plate and 150 μL of 2,2′-Bipyridine solution was added. The plate was read on a spectrophotometer at 510 nm. A linear standard curve was created by plotting the absorbance against phytic acid concentration. The total concentration was determined by using the linear equation from the standard curve: *y* = −0.0015*x* + 0.7468. For the purpose of this paper, phytic acid was expressed as phytic acid phosphorus. However, phytic acid phosphorus can be converted to phytic acid by multiplying the value by 3.548 [32].

### 2.8. Shelf-Life Tests

Two tests were conducted to assess the shelf life of the food formulations. In the first, the stability of the product was assessed at 4 °C for eight weeks. Samples were taken every week and analyzed for pH, acidity and viable counts. In the second test, a non-optimal condition was simulated and products were stored at room temperature (22 °C) for 5 days. Samples were taken every 12 h and analyzed for pH, acidity and viable counts.

### 2.9. Statistical Analysis

A one way repeated measures analysis of variance (ANOVA) was used to evaluate variances in the hedonic scores of the sensory analysis. A two way repeated measures ANOVA was used to determine variances in data amongst the water and milk based millet products when sweetened with sucrose and honey. An XY data graph was used to analyze four samples including a control, from the millet test, comparing acidity and pH along with viable counts of *L. rhamnosus* GR-1. All statistical analyses were determined by GraphPad Prism 7.00 (San Diego, CA, USA).

## 3. Results

### 3.1. Fiti Sachet Used to Ferment Milk

#### Cell Growth and Acidity Profile

**Figure 3:**
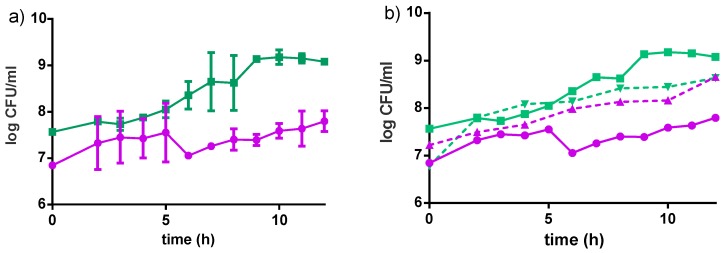
Growth curves of *L. rhamnosus* GR-1 (●) and *S. thermophilus* C106 (■) populations when grown in (**a**) consortium (straight line); or (**b**) as single culture (dotted line) in milk. Fermentations were carried out at 40 °C for 12 h. Curves show mean values and error bars based on two independent replicates.

The Fiti sachet bacteria (10^9^ each of *L. rhamnosus* GR-1 and *S. thermophilus*) grew in milk and altered the pH and acidity of the substrate (Figure 3). *S. thermophilus* showed a 2 log CFU/mL increase in cell density during the 12 h fermentation, while an increase of 1 log cfu/mL was observed for *L. rhamnosus* GR-1. The initial pH of the milk was stable at 6.4 in the first 4 h of incubation, then dropped to pH 5.3 within a subsequent 3 h incubation, and continuously decreased until a final value of pH 4.2 was reached by the end of the fermentation. The decrease in pH corresponded to an increase in acidity of 17.3°N, which is a measure of the lactic acid content. The same parameters were measured for the single cultures of *L. rhamnosus* GR-1 and *S. thermophilus* in milk (12 h incubation, 40 °C) and results are shown in Figure 3. When *L. rhamnosus* GR-1 grew alone in milk, a decrease of only one pH unit was measured, with a final value of 5.66, while a final pH of 4.6 was measured with a single culture of *S. thermophilus*. Following the same pattern, the single culture of *L. rhamnosus* GR-1 increased the acidity of 6.15°N, while the single culture of *S*. *thermophilus* increased the acidity of 15.2°N.

When measuring the specific growth rate of the two bacteria in media (M17 for *S. thermophilus*, modified MRS for *L*. *rhamnosus*) enriched with 0.5% of different carbon sources, *L. rhamnosus* GR-1 showed the highest growth in dextrose, galactose, and fructose. Lactose was utilized, however it was not the most suitable carbon source for this strain, while maltose, melibiose, raffinose, and sucrose were not fermented (Table 1). *S. thermophilus* showed the highest growth in sucrose and lactose, and lower in galactose and dextrose. Some growth occurred in melibiose, raffinose, and fructose. Maltose was the only carbon source for this strain that was not utilized.

### 3.2. Fiti Sachet in Millet Media

#### 3.2.1. Bacterial Growth in Millet

**Figure 4:**
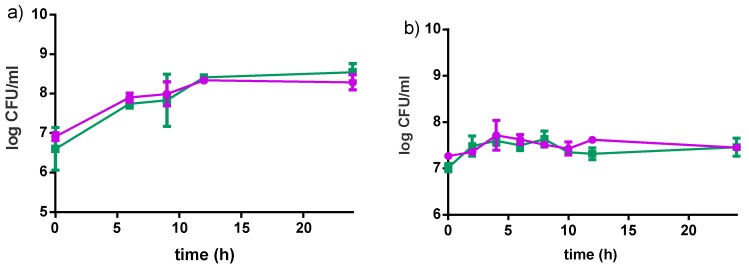
Growth curves of *L. rhamnosus* GR-1 (●) and *S. thermophilus* C106 (■) as a consortium in the Fiti sachet (**a**), or as single cultures (**b**) in 8% pre-treated millet in water dispersion (60 min pre-treatment at 90–95 °C). Fermentation was carried out at 40 °C for 24 h. Mean values and error bars are based on two independent replicates.

The strains grew better individually in water dispersion than when incubated together (Figure 4). Both strains reached higher cell populations when grown in milk-based formulations rather than in water-based formulations (Table 2). An increase in about 2 log CFU/mL was observed for *L. rhamnosus* GR-1 when grown in milk-based media after 12 h of incubation at 40 °C, while an increase in about 1.5 log CFU/mL was observed in the water-based product, in the same conditions. For *S. thermophilus*, an increase of about 3 log CFU/mL was observed in the milk-based formulations, while an increase of 2 log CFU/mL was observed in water-based media after 12 h incubation at 40 °C. The Fiti sachet contains a 1:1 ratio of the two bacteria, which were therefore present in the same ratio in all media at time zero of the fermentation. No significant difference in viable cells was observed for either strain when grown in medium enriched with honey, rather than sucrose. Moreover, viable counts of *L. rhamnosus* GR-1 and *S. thermophilus* in the milk-based (4% millet, 5% sucrose) and water-based (8% millet, 5% sucrose) products were determined during the fermentation (12 h, 40 °C) and refrigerated storage (4 °C) for eight weeks.

**Figure 5:**
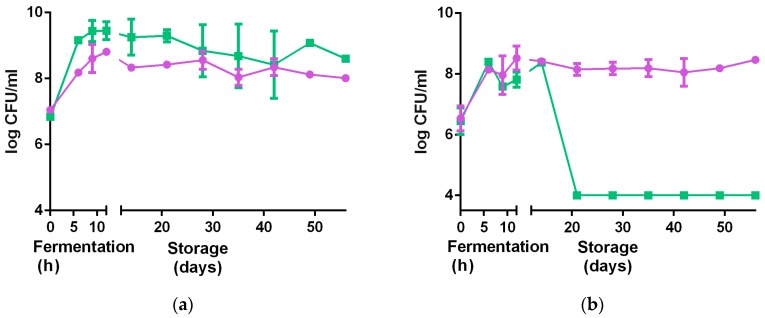
Changes in viable bacteria counts of *L. rhamnosus* GR-1 (●) and *S. thermophilus* C106 (■) during fermentation (12 h) and refrigerated storage (56 days). (**a**) 4% millet in milk with 5% sucrose; (**b**) 10% millet in water with 5% sucrose. Error bars represent standard deviations based on two independent replicates.

Results are shown in Figure 5. *L. rhamnosus* GR-1 decreased of 1 log CFU/mL throughout the entire storage period in the milk-based product, and a slight decrease in the water-based product. A similar trend was observed for *S. thermophilus* in the milk-based product (1 log CFU/mL reduction in eight weeks), while a drastic reduction of 5 log CFU/mL was observed in the water-based product. This significant reduction was likely the result of the low buffering capacity of the water-based formulations, together with the decrease in pH due to organic acids produced from the two bacteria.

#### 3.2.2. Acidification Profile

**Figure 6:**
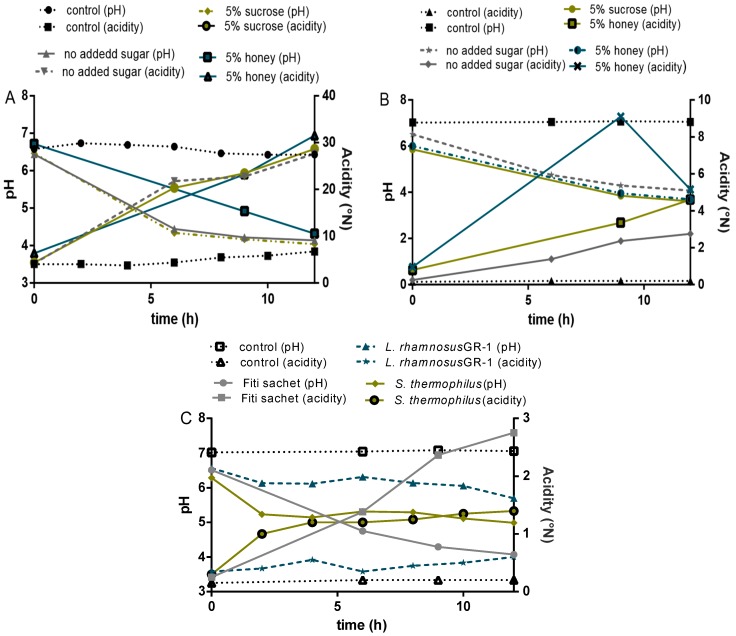
(**A**) 4% millet in milk; (control: milk); (**B**) 8% millet in water; (**C**) single cultures in milk.

The pH levels and acidification patterns in the final products depended on the strains used and whether the formulation was milk- or water-based. A decrease in pH was observed in all formulations, and followed a pattern similar to other milk and cereal-based fermented products reported in the literature. Acidification profiles showed two different trends for water and milk-based products (Figure 6). Two phases of acidification were distinguished in the milk-based products: the first one occurring during the first 6 h when the pH decreased from 6.5 ± 0.1 to 4.4 ± 0.4, followed by a second phase of slower acidification (from 6 to 12 h) that resulted in a final pH of 3.9 ± 0.2. Reduction in pH occurred at a faster rate in the water-based formulations, compared to the milk-based, despite the fact that the milk-based contained six to 10 times the amount of lactic acid. Similar trends were reported by Helland et al. [34] while testing the probiotic fermentation of milk- and water-based cereal puddings.

To determine the acid production, titratable acidity was measured during fermentation and storage, expressed in millimoles of NaOH per 100 g yogurt/milk [35]. An initial average pH value of 6.5 ± 0.1 was recorded in all water and milk-based formulations fermented with Fiti sachet or a single culture of *L. rhamnosus* GR-1. Interestingly, samples fermented with a single culture of *S. thermophilus* showed a slightly lower initial pH (6.3 ± 0.1) than the average of this study when grown in millet-formulations, and a slightly higher pH (6.9 ± 0.1) when grown in milk-formulations. An initial acidity of about 0.3 was recorded for the water-based formulations and values of around 5 for the milk-based, which confirms the buffering capacity of the milk. Acidity steadily increased in both cases, but by significantly different rates. In fact, final values of about 4.5 ± 1.0 were reported for water-based products, while values of about 3.0 ± 5.0 were reported for the milk-based products.

#### 3.2.3. Organic Acids and Carbohydrate Analysis

Chromatography techniques were used to identify the main compounds involved in matrix changes during fermentation. The gas chromatography results were aligned and normalized to the internal standard ribitol. A principal component analysis was performed in order to identify the compounds driving the changes between data sets at the beginning, and at the end of the fermentation. A reduction in sugars and an increase in organic acids was observed as a general trend. For the fermentation in water-based formulations (Table 3), d-glucose, sucrose and d-mannose were the most abundant sugars at time zero and were almost totally depleted after 12 h of fermentation, except for the sucrose (only 2/3 consumed). Lactic acid was the most abundant acid produced and a small increase in acetic acid was also detected. Other compounds such as 1,2-butandiol, a starch degradation product, slightly increased during the fermentation. Furthermore, a small increase in other beneficial compounds such as tryptophan were observed during the fermentation (data not shown). For the fermentation in milk-based formulations, a decrease in d-lactose (0.268 g/L to 0.199 g/L), maltose (1.190 g/L to 1.065 g/L), and citrate (0.706 g/L to 0.739 g/L) was observing during the fermentation. This occurred with an increase in glycerol (0.659 g/L to 1.639 g/L), d-galactose (0.815 g/L to 1.694 g/L), and lactic acid (0.160 g/L to 1.650 g/L). Phytic acid concentrations of the millet products were determined using the modified colorimetric method of Haug and Lantzsch [31]. In this two-step method, the phytic acid is precipitated with an acidic iron (III) solution of known iron concentration, and the decrease in iron in the supernatant is a measure of the phytic acid content. The average results of three phytic acid determination experiments showed a decrease in phytic acid as the fermentation time increased with time zero having the highest concentration of phytic acid. Therefore, an increase in fermentation resulted in more free iron, which would increase the bioavailability of this and other minerals.

#### 3.2.4. Sensory Evaluation

The organoleptic properties of the milk-based formulations were comparable to yogurt, with respect to structure and flavor. Yogurt is a fermented milk product with a distinct flavor, such that it has a smooth viscous gel structure and a slightly sour taste [36]. Therefore, yogurt should be evaluated according to appearance, flavor, texture, and overall quality [37]. The sensory test presented to all participants consisted of two parts. The selected samples for the sensory analysis are shown in Table 4. In the first part of the sensory analysis, participants were asked to rank three different products using the hedonic scale provided. The second part, involved a comparison test among the same samples (4% millet in milk + 5% sucrose) but with differing pre-treatment (30 min vs. 60 min) and fermentation times (9 h vs. 12 h). Overall, 4% millet in milk, with a 60 min pre-treatment time and fermented for 12 h was preferred. Sample 214 received the lowest score among Africans and an improved score from panel test A.

Table 5 depicts that percentage of participants who consumed yogurt, porridge, milk based porridge and water based porridge each week. From these findings, we can conclude that Panel Test A preferred water based porridge whereas Panel Test B preferred milk based porridge. Furthermore, yogurt was consumed more often compared to porridge in Panel Test A, whereas in Panel Test B, both yogurt and porridge were consumed equally as often.

**Figure 7:**
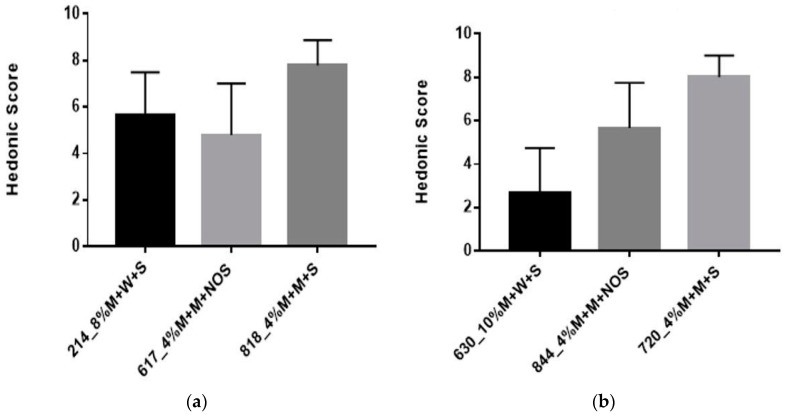
(**a**) Results from the hedonic test Panel Test A; (**b**) Results from the hedonic test Panel Test B. M = millet, W = water, S = sugar, NOS = no sugar.

The results from the hedonic score from Panel Test A (Figure 7a) indicated an overall preference for #818 which had a mean score of eight. There was a significant difference between each sample (*p* < 0.0001), as the milk-based millet was preferred over water-based millet. The results from the hedonic score from Panel Test B (Figure 7b) indicated an overall preference for sample 720, which had a mean score of eight. From Test A and B, samples 818 and 720 respectively were the same. There was no significant difference amongst the three samples (630,720,844) (*p* = 0.1245).

#### 3.2.5. Stability at Non-Optimal Conditions

**Figure 8:**
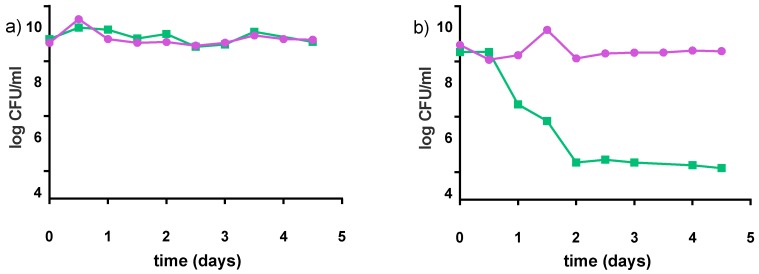
Survival of *S. thermophilus* C106 (■) and *L. rhamnosus* GR-1 (●) during storage at 22 °C for five days; (**a**) milk-based formulation (4% pre-treated millet in milk, 5% sucrose); (**b**) water-based formulation (8% pre-treated millet in water, 5% sucrose). Mean values are based on two independent replicates.

The preferred storage condition for our products was 4 °C. However, refrigeration is not often practical in some regions of Uganda, Kenyan and Tanzanian communities where ambient temperatures are often 21–25 °C. Accordingly, the stability of the products was tested at 22 °C for 5 days (which based upon our experience would be the longest these yogurts stay in community kitchens before purchase). Samples were analyzed with regards to pH and acidity, viable counts (Figure 8) and growth of spoilage bacteria. The pH values slightly decreased over five days by 0.2 points for the milk-based formulations, and remained almost constant for water-based formulations. Both *S. thermophilus* and *L. rhamnosus* GR-1 showed stability in the viability of the milk matrix. A different pattern was observed for *S. thermophilus* in water matrix, where the viable counts decreased by almost 4 log cfu/mL within the first two days of storage.

## 4. Discussion

### 4.1. Fermentation Ability of Fiti Sachet Bacteria

In this study, a probiotic Fiti sachet (one gram of a freeze-dried consortium of *L. rhamnosus* GR-1 and *S. thermophilus* C106), which is being used in sub-Saharan Africa, was shown to ferment milk and millet. The addition of a Fiti sachet to one liter of milk resulted in a decrease in pH by 2 units, and an increase in acidity by 17.3°N. These values are in accordance with the values generally observed in yogurt, which reduces contaminants and increase food security. However, the Fiti sachet requires a longer incubation time (12 h), compared to traditional yogurt, a consequence perhaps of not having *L. delbrueckii* subsp. *bulgaricus* present. A similar acidification profile was observed during the fermentation of milk with the probiotic consortium of *S. thermophilus* C106 (the same strains used in Fiti sachets) and *L. rhamnosus* yoba 2012 [26].

Interestingly, in our study, both cultures did not appear to benefit from growing as a mixed culture in milk. The *S. thermophilus* C106 population increased by 1.5 log CFU/mL in the consortium and 1.89 ± 0.3 when grown as a single culture. *L. rhamnosus* GR-1 population increased by 0.95 ± 0.2 log CFU/mL in consortium and of 1.41 ± 0.1 log CFU/mL when grown as a single culture. The proteolytic strain *S. thermophilus* C106 present in Fiti sachet differs from the *S. thermophilus* used in the traditional yogurt starter bacteria. As described by Kort et al. [26], this strain does not need the metabolic activity (proto-cooperation) of a *Lactobacillus* strain, as it is able to synthesize all essential amino acids indispensable for growth in milk, and to degrade casein and lactose. Its combination with *L. rhamnosus* Yoba 2012 in the probiotic Yoba yogurt was described as necessary for the growth of the probiotic strain in milk, as it is unable to degrade casein and lactose.

A combined analysis of the genome of *S. thermophilus* C106 and *L. rhamnosus* Yoba 2012 suggested that *Streptococcus* provides folic acid, galactose, glycerol, peptides, succinate, and xanthine/guanine to the prebiotic strain, when grown in milk [26]. The only cooperation provided by the probiotic strain could be the galactose utilization, which avoids product inhibition of *S. thermophilus*. A similar interaction possibly occurs between *S. thermophilus* C106 and *L. rhamnosus* GR-1. However, in our study, both bacteria showed a higher viable count at the end of the fermentation when grown alone in milk, instead of growing as a consortium.

The distal urethral isolate *L. rhamnosus* GR-1 shares most but not all of its genome with *L. rhamnosus* GG. We observed that in contrast to *L. rhamnosus* yoba 2012, *L. rhamnosus* GR-1 is able to metabolize lactose, and this metabolic difference might explain why this strain was able to reach similar viable counts when grown as a single culture, or as a mixed culture in consortium with *S. thermophilus* C106 in milk. Douillard et al. [38] compared the genome of 100 species of *L. rhamnosus* isolated from a large variety of ecological niches with the genome of *L. rhamnosus* GG. They observed that carbohydrate transport and metabolism genes were located in highly variable regions of the *L. rhamnosus* genome, and were not part of the core genome (shared gene set) of the *L. rhamnosus* species. In the *L. rhamnosus* GG metabolic pathway, d-lactose utilization is partially altered and therefore not functional, preventing the strain from metabolizing lactose. However, other isolates of *L. rhamnosus* have these genes intact and can therefore ferment lactose, and this appears to be the case with *L. rhamnosus* GR-1.

In contrast to numerous types of LAB, *S. thermophilus* is unable to ferment a large number of sugars [39]. It is well adapted to growth in milk and to use lactose as a primary carbon and energy source, of which only the glucose moiety is used, while the galactose moiety is excreted in the growth medium. According to Poolman and van de Bogaard [40,41], *S. thermophilus* strains are only able to use glucose, lactose, sucrose and fructose as carbon sources. When able to use glucose and fructose, the growth rates are several-fold lower than the ones in lactose or sucrose, which are readily metabolized. This is in accordance with the most suitable carbon sources identified for *S. thermophilus* C106 in our study, which indeed were sucrose and lactose. However, sufficient growth was unusually observed for this strain on galactose, which is normally not fermented by most *S. thermophilus* strains used as starter cultures [42].

Vaughan et al. [43] found that strain CNRZ302 of *S. thermophilus* contained structurally intact genes for the Leloir pathway enzymes, which are responsible for d-galactose catabolism, where the upregulation of the *gal* gene cluster promoter seems to be sufficient for a galactose-fermenting phenotype of *S. thermophilus*. In fact, bacteria actively regulate their metabolism according to the availability of carbon sources in the growth medium, and utilize mechanisms to allow the cell to selectively utilize the most favorable carbon source present in the growth medium, and to repress operons encoding genes for uptake and metabolism of less favorable sugars. This might explain, in our study, the ability of *S. thermophilus* C106 to utilize galactose in the absence of other favorable carbohydrates (Table 1).

The addition of a Fiti sachet to one liter of millet-based matrix, resulted in a pH drop to a final value of 3.9 ± 0.2 for both water and milk-based formulations. However, a reduction in pH occurred at a faster rate in the water-based formulations, compared to the milk-based formulations, despite the fact that the milk-based contained six to 10 times the amount of lactic acid. It was observed that LAB growth was enhanced in media containing pre-treated millet rather than milk, and a significant amount of lactic acid was produced (0.6 g/L). Decrease in pH and an increase in lactic acid followed the same trend, as reported by Rathore et al. [44]. Probiotic fermentation of single and mixed cereal substrates of malt and barley increased the production of lactic acid compared to the fermentation reported by Helland et al. [45] in maize porridge with added malted barley.

The decrease in pH values may be due to the low buffering capacity of the water-based products, and the low final pH of these formulations combined with the production of antimicrobial compounds by LAB (lactic acid and possibly bacteriocins). These alone could be a significant growth-limiting factor for food-borne pathogens, indicated by a decrease in viable cell counts during storage of both strains, particularly for *S. thermophilus* C106. Helland et al. [45] suggested that supplementation of ingredients that enhance the buffering capacity of the formulations would probably also increase fermentation time and survival, and this trend was observed in our study [34]. In fact, the addition of milk to the water-based formulations caused a delay in the decrease of the pH, which slowly lowered over the first 9 h of fermentation, and only reached a final pH value of about 3.9 in the end of the fermentation (12 h). The addition of milk (buffering agent) also increased the bacterial survival rate during storage [34]. This was particularly evidenced by *S. thermophilus* C106, which almost disappeared in our detected values (>1000 UFC/mL) after three weeks of refrigerated storage when grown in water-based medium. There was only a decrease of 0.5 log CFU/mL over eight weeks of refrigerated storage, when grown in a milk-based medium. A similar trend was observed for *S. thermophilus* C106 during storage at room temperature (22 °C) for five days. The genus *Streptococcus* is known to be more sensitive to acidic environments than *Lactobacillus*. Therefore, it is possible that *S. thermophilus* C106 is affected by the acidic environment of the water-based formulations during storage. Overall, the amount of phytic acid lowered with an increase in fermentation time. Since phytate binds to minerals such as iron and zinc, a decrease in this organic acid would increase the bioavailability of these minerals in the host.

### 4.2. Product Development

The preparation of traditional fermented cereal foods often includes a soaking step, which softens grains and makes them easier to crush or wet mill, from which hulls, bran particles, and other substances can be removed by sieving procedures. After fermentation, the slurry is generally boiled with an appropriate amount of water so that gelatinization of starch occurs, and the product becomes more digestible with a more palatable texture [46]. However, this step kills all LAB present in the fermented cereal, and is therefore unwanted for probiotic foods, where beneficial bacteria must be alive inside the final product. Both preparation procedures used here, retained bacterial viability and included a gelatinizing heat treatment step prior to the inoculation of the bacteria.

The chemical composition of cereal grains consists of a high carbohydrate content (60–70%), proteins (8–11%), lipids (2–4%) and minerals (1–3%) [47]. The principal carbohydrate components of cereal grains are starch, water-soluble or water-insoluble components of dietary fiber, and several free sugars, such as glucose, glycerol, stachyose, xylose, fructose, maltose, sucrose, and arabinose. In general, cereals are suitable substrates for the growth of LAB and human-derived probiotic strains [14]. Heating of cereal grains induces a series of structural changes, namely gelatinization, where amylose dissolves, leaches, in water and increases the viscosity of the suspension.

An objective of this study was to achieve cell concentrations between 10^6^ and 10^7^ CFU/mL for the probiotic strain, which would ensure an adequate cell concentration at the time of consumption (based on a 100 mL daily intake of the product). Moreover, high viable counts of *S. thermophilus* are necessary to obtain the desired acid production and reduction in pH, which prevents product contamination, and affects the shelf-life and organoleptic properties of the final product. These values were already reached after 9 h of fermentation, and 10^8^–10^9^ CFU/mL by the end of the incubation period (12 h, 40 °C). The highest values were obtained with flour based formulations, probably due to a significantly higher percentage of millet in the medium. These values are in accordance with the cell populations obtained from fermentation (12 h, 37 °C) of milk-based maize and rice puddings (8–9.1 log CFU/mL) with probiotic strains *Lactobacillus acidophilus* La5, *Bifidobacterium animalis* Bb12, *L. acidophilus* NCIMB 701748 and *L. rhamnosus* GG [34]. In that same study, *L. rhamnosus* GG was the only strain showing substantial growth in both milk and water-based puddings, reaching values of 9.1 and 8 log CFU/mL at the end of the fermentation [34].

Since all experiments were performed under uncontrolled pH conditions, the rapid drop in pH in mixed-culture fermentations was due to accumulation of lactic acid produced via the metabolic pathways, mainly from *S. thermophilus*. This species is obligately homofermentative and almost exclusively produces lactic acid as an end-product from glycolysis of carbohydrates via the Embden-Meyerhof pathway [46]. The observed decrease in glucose during fermentation of millet in water formulation closely correlated with the increase in lactic acid. This indicates that *S. thermophilus* C106 is mainly responsible for the decrease in pH of our formulations. This is in agreement with the small decrease in pH reported during the growth of a single culture of *L. rhamnosus* GR-1 in the same medium (Figure 3c). However, Helland et al. [34] reported an efficient metabolic conversion of glucose into lactic acid, acetoin and diacetyl from *L. rhamnosus* GG in fermented milk and water-based cereal puddings. In both studies, *L. rhamnosus* GG produced 9500–9800 mg/kg (milk-based) and 4000 mg/kg (water-based) lactic acid at the end of fermentation (24 h) and a decrease in pH from 5.8 to 3.1–3.7 in water-based formulations. This drop in pH was not observed in our study when *L. rhamnosus* GR-1 was cultivated as a single culture in water-based formulations, where values of 5.7 and 5.3 ± 0.2 were measured after 12 and 24 h of fermentation. On the other hand, a single culture of *L. rhamnosus* GR-1 caused a slower but significant drop in pH in milk media, with values of 5.6 ± 0.2 and 3.7 after 12 and 24 h of incubation. *L. rhamnosus* GR-1 is a facultatively heterofermentative LAB [48] that can use the pentose phosphate pathway for utilizing pentoses, resulting in production of lactic acid and ethanol/acetate. Therefore, *L. rhamnosus* GR-1 was likely responsible for the increase in acetic acid observed in our study (Table 3). Lactate and acetate represent important flavor compounds in fermented cereals [49], with acetate being reported as a flavor enhancer [50].

Honey is composed of monosaccharides which can be easily degraded by bacteria, compared to disaccharides. The lack of effect that honey had on the Fiti strains might be related to the sucrose preference of *S. thermophilus* C106, rather than fructose and dextrose as a carbon source. This assumes there is cooperation between the two bacteria, as described by Kort et al. [26]. Growth of *S. thermophilus* C106 is necessary for the propagation of *L. rhamnosus* GR-1, as it requires some of the metabolites produced by this bacterium [27]. Accordingly, *L. rhamnosus* GR-1 cannot reach a higher viable cell count, when compared to the substrate with sucrose, although this strain showed a preference for fructose and dextrose, rather than sucrose, as a carbon-source (Table 1).

The cooperation between the two bacteria was necessary in order to obtain a yogurt-like probiotic product. When grown alone in milk, *L. rhamnosus* GR-1 produced insufficient decrease in pH and no viscous texture at the end of the fermentation (12 h). A similar cooperation appeared to take place in the millet-substrate, as there was no significant decrease in pH observed at the end of the fermentation, when *L. rhamnosus* GR-1 was grown alone in 8% millet in water dispersion. All tested recipes fermented with Fiti sachet as consortium led to a product which could be defined as probiotic for the number of viable cells of the probiotic strain *L. rhamnosus* GR-1.

Consumer acceptance varied for the different formulations. The milk-based recipes had a much higher score based on the hedonic test, than the water-based products, particularly for the panel tested by Africans. Overall, the preferred recipe was 4% millet in milk, with pre-treatment of 60 min at 90–95 °C for the millet, then addition of 5% sucrose and incubation for 12 h at 40 °C. This formulation proved to remain stable, in terms of viable probiotic cells, over eight weeks of refrigerated storage and five days of storage at room temperature. When milk is not available or affordable for the producers, assuming a small-scale production in kitchens in sub-Saharan Africa, a replacement of 50% of the milk with water (that has been boiled and cooled or from a non-contaminated source) can be done without compromising the sensory qualities of the product.

Further investigation of the sensory acceptance of the flour-based formulations would be worthwhile with a larger pool of subjects, and an aim of utilizing fermented millet as a vehicle for delivering a novel probiotic food. Nutritional information was not analyzed before and after fermentation, and this would have been informative in terms of the micro- and macronutrient composition of the various products, especially relating to phytic acid concentrations.

## 5. Conclusions

This study showed that a Fiti sachet (one gram freeze-dried consortium of *L. rhamnosus* GR-1 and *S. thermophilus* C106) is capable of producing novel, tasty millet-based fermented probiotic products, with a considerable amount of lactic acid produced when milk is included. Pure culture fermentations of the two individual strains led to similar amounts of cell populations, but the decrease in pH was considerably lower, thereby likely increasing food safety as well as the sensory properties of the product. Low pH seems to be the main limiting factor for microbial growth, as fermentable sugars were still detected at the end of the fermentation. Bacterial viability was sustained during two months of refrigerated storage and five days of room temperature storage. The sensory evaluation showed a preference for a formulation that had 4% millet in milk, with a pre-treatment of 60 min of the millet, addition of 5% sugar and 12 h of fermentation at 40 °C. This is easily reproducible in community kitchens in sub-Saharan Africa. Feedback from African participants revealed that an adaptation of the recipe to reduce milk and sweeteners, which can be unaffordable in some rural areas of sub-Saharan Africa, would not compromise the acceptance of the product in these areas. Furthermore, a good acceptance of the flour based recipe was suggested by the same African panelists, because of its similarity to other locally available traditional fermented beverages and foods.

In other regions of the world, food types such as rice, lentils used in India [51], and millet-based Teff used in flat bread Injera in Ethiopia [52] may also be worthy of testing to create added-value probiotics, but as heat kills the bacterial strains, the advantage of the fermented products described here is that the product has good taste and texture, and does not need to be consumed heated.

## Figures and Tables

**Table 1:** Maximum growth rate (µ_max_) of *L. rhamnosus* GR-1 and *S. thermophilus* C106 in media (M17, modified MRS) enriched with 0.5% of different carbon sources.

Carbon Source	*L. rhamnosus* GR-1 (µ_max_)	*S. thermophilus* C106 (µ_max_)
Dextrose	0.4954	0.2669
Fructose	0.4045	0.1136
Galactose	0.4445	0.4251
Lactose	0.2311	0.8770
Maltose	0.0607	0.0959
Melibiose	0.0330	0.1397
Raffinose	0.0909	0.1348
Sucrose	0.0678	1.7046
No C-source	0.1029	0.0948

**Table 2:** Viable counts (cfu/mL) of *L. rhamnosus* GR-1 (L. GR-1) and *S. thermophilus* C106 (S. therm.) in various media after 0, 9 and 12 h of fermentation at 40 °C. Note, 9.33 E + 06 = 9.33 × 10^6^.

Recipe	*T* = 0 h	*T* = 0 h	*T* = 9 h	*T* = 9 h	*T* = 12 h	*T* = 12 h
	L. GR-1	S. therm.	L. GR-1	S. therm.	L. GR-1	S. therm.
*Milk-Based*						
4% millet, no added sugar	9.33 E + 06	5.67 E + 06	4.00 E + 08	2.00 E + 09	6.70 E + 08	2.70 E + 09
4% millet, 5% sucrose	6.50 E + 06	5.33 E + 06	2.50 E + 08	1.50 E + 09	9.00 E + 08	1.67 E + 09
4% millet, 5% honey	2.25 E + 06	2.00 E + 06	2.10 E + 08	9.70 E + 08	3.50 E + 08	1.40 E + 09
Flour, 3% sucrose	3.67 E + 06	6.33 E + 06	-	-	7.67 E + 09	3.67 E + 10
Flour, 50% water + 50% milk	3.67 E + 06	6.00 E + 06	-	-	8.33 E + 09	4.67 E + 10
*Water-Based*						
8% millet, 5% sucrose	7.67 E + 06	4.67 E + 06	-	-	9.33 E + 07	3.30 E + 07
8% millet, no added sugar	1.22 E + 07	7.88 E + 06	2.19 E + 08	2.52 E + 08	2.29 E + 08	3.62 E + 08

**Table 3:** Loading plot: compounds driving the changes during fermentation (T = 40 °C, 12 h) of 8% pre-treated millet (1 h, 85 °C) in water.

Compound	*T* = 0 h (µg/mL)	*T* = 12 h (µg/mL)
d-Glucose	986.2	52.6
Sucrose	847.8	341.9
d-Mannose	667.4	Not detected
Lactic Acid	99.0	806.8
Acetic Acid	2.3	3.2
1,2-Butandiol	2.1	3.2

**Table 4:** Sample codes for sensory analysis.

Sample Code	Sample Composition
214	8% millet in water, 60 min pre-treatment, 5% sucrose
630	10% millet in water, 60 min pre-treatment, 3% sucrose
818/720	4% millet in milk, 60 min pre-treatment, 5% sucrose, 12 h fermentation
617/844	4% millet in milk, 60 min pre-treatment, 12 h fermentation
M4	4% millet in milk, 30 min pre-treatment, 5% sucrose, 12 h fermentation
M5	4% millet in milk, 60 min pre-treatment, 5% sucrose, 9 h fermentation

**Table 5:** Composition of panel test A and B.

	Panel Test A	Panel Test B
% yogurt consumed/week	89.5	67
% porridge consumed/week	47.4	67
% milk based porridge consumed/week	36.8	100
% water based porridge consumed/week	42.1	33
